# Genes encoding putative bicarbonate transporters as a missing molecular link between molt and mineralization in crustaceans

**DOI:** 10.1038/s41598-021-91155-w

**Published:** 2021-06-03

**Authors:** Shai Abehsera, Shmuel Bentov, Xuguang Li, Simy Weil, Rivka Manor, Shahar Sagi, Shihao Li, Fuhua Li, Isam Khalaila, Eliahu D. Aflalo, Amir Sagi

**Affiliations:** 1 Department of Life Sciences, Ben-Gurion University of the Negev, P.O. Box 653, 8410501 Beer Sheva, Israel; 2 The National Institute for Biotechnology in the Negev, Ben-Gurion University of the Negev, Beer-Sheva, Israel; 3 Freshwater Fisheries Research Institute of Jiangsu Province, Nanjing, People’s Republic of China; 4 Key Laboratory of Experimental Marine Biology, Institute of Oceanology, Chinese Academy of Sciences, Qingdao, People’s Republic of China; 5 Department of Biotechnology Engineering, Ben-Gurion University of the Negev, Beer-Sheva, Israel; 6 Department of Life Sciences, Achva Academic College, Arugot, Israel

**Keywords:** Biochemistry, Physiology

## Abstract

During their life, crustaceans undergo several molts, which if theoretically compared to the human body would be equivalent to replacing all bones at a single event. Such a dramatic repetitive event is coupled to unique molecular mechanisms of mineralization so far mostly unknown. Unlike human bone mineralized with calcium phosphate, the crustacean exoskeleton is mineralized mainly by calcium carbonate. Crustacean growth thus necessitates well-timed mobilization of bicarbonate to specific extracellular sites of biomineralization at distinct molt cycle stages. Here, by looking at the crayfish *Cherax quadricarinatus* at different molting stages, we suggest that the mechanisms of bicarbonate ion transport for mineralization in crustaceans involve the SLC4 family of transporters and that these proteins play a key role in the tight coupling between molt cycle events and mineral deposition. This discovery of putative bicarbonate transporters in a pancrustacean with functional genomic evidence from genes encoding the SLC4 family—mostly known for their role in pH control—is discussed in the context of the evolution of calcium carbonate biomineralization.

## Introduction

Biomineralization, the process by which living organisms deposit minerals, is widespread across the animal kingdom^1^, including crustacean species, which are characterized by a rigid mineralized outer skeleton^2^. In this evolutionary group of arthropods^3^, the exoskeleton is periodically shed (and subsequently regenerated) to enable growth. A molting crustacean has a soft shell, which exposes the animal to risks of predation and renders it unable to move, since contraction of the muscles is contingent on the rigidity of the exoskeleton. The resulting extreme vulnerability has led to the evolution of a variety of mineralization strategies in these animals^4,5^. Indeed, Lowenstam and Weiner (1989) have dubbed the crustaceans “the champions of mineral mobilization and deposition in the animal kingdom”. In these animals, mineralization occurs mainly through the deposition in the cuticle of calcium carbonate^6,7^ in the form of various polymorphs and different levels of hydration and is therefore generally referred to throughout the article as calcium carbonate. The crustacean biomineralization process is linked to a molt cycle comprising four main stages that in our study organism are reflected in changes in the cuticle and the transient calcium storage organ, the gastrolith: inter-molt, an intermediate stage during which no molt activity occurs both in the cuticle and the gastrolith; pre-molt, a stage characterized by the storage of calcium in gastroliths in preparation for molting and by the formation of a new non-mineralized cuticle underneath the existing exoskeleton; ecdysis, the act of shedding the old cuticle concomitantly with the collapse of the gastrolith into the stomach; and post-molt, a stage in which the calcium stored at pre-molt is released by gastrolith digestion and utilized for mineralization and maturation of the new cuticle^6,8,9^.

Similarly to crustaceans, numerous other biomineralizing organisms utilize different forms of carbonate for hardening of their skeleton; these organisms span a wide range of taxonomic groups, from unicellular organisms (such as coccolithophores^10^), sponges^11^, and stony corals^12^ to vertebrates. In vertebrates bicarbonate ions, being the most abundant ions after calcium and phosphate, play a central role in bone mineralization^13,14^ via the pH regulation necessary for the control over such processes^15–19^. The transport of bicarbonate ions as part of the biomineralization process in these non-crustacean organisms is hypothesized to be facilitated by members of the SLC4 family of bicarbonate transporters^18,20–27^ although it is important to note that these studies have only relied on gene upregulation associated with mineralization and not on direct evidence such as knock out experiments for the involvement of the SLC4’s in biomineralization. Among the 10 known members of the SLC4 family of transport proteins^28^, the best studied—by virtue of its role as the regulator of cell pH in red blood cells—is SLC4A1 (also known as AE1 or band 3^28^), an electroneutral antiporter of HCO_3_^−^ and Cl^–^^29,30^. Another member of the SLC4 family that is also involved in pH regulation in various tissues^31^ is the Na^+^-coupled electroneutral symporter of bicarbonate, SLC4A7 (also known as NBCn1, NBC2 or NBC3). On the basis of the above-described roles for SLC4 transporters in the regulation of pH and the supply of ions for the biomineralization of calcium minerals in non-crustacean organisms, it seemed likely that SLC4 transporters would also be involved in crustacean biomineralization, even though such an involvement had not been reported at the time of this study.

## Results and discussion

### Discovering ion-transport proteins in a crustacean

**Figure 1:**
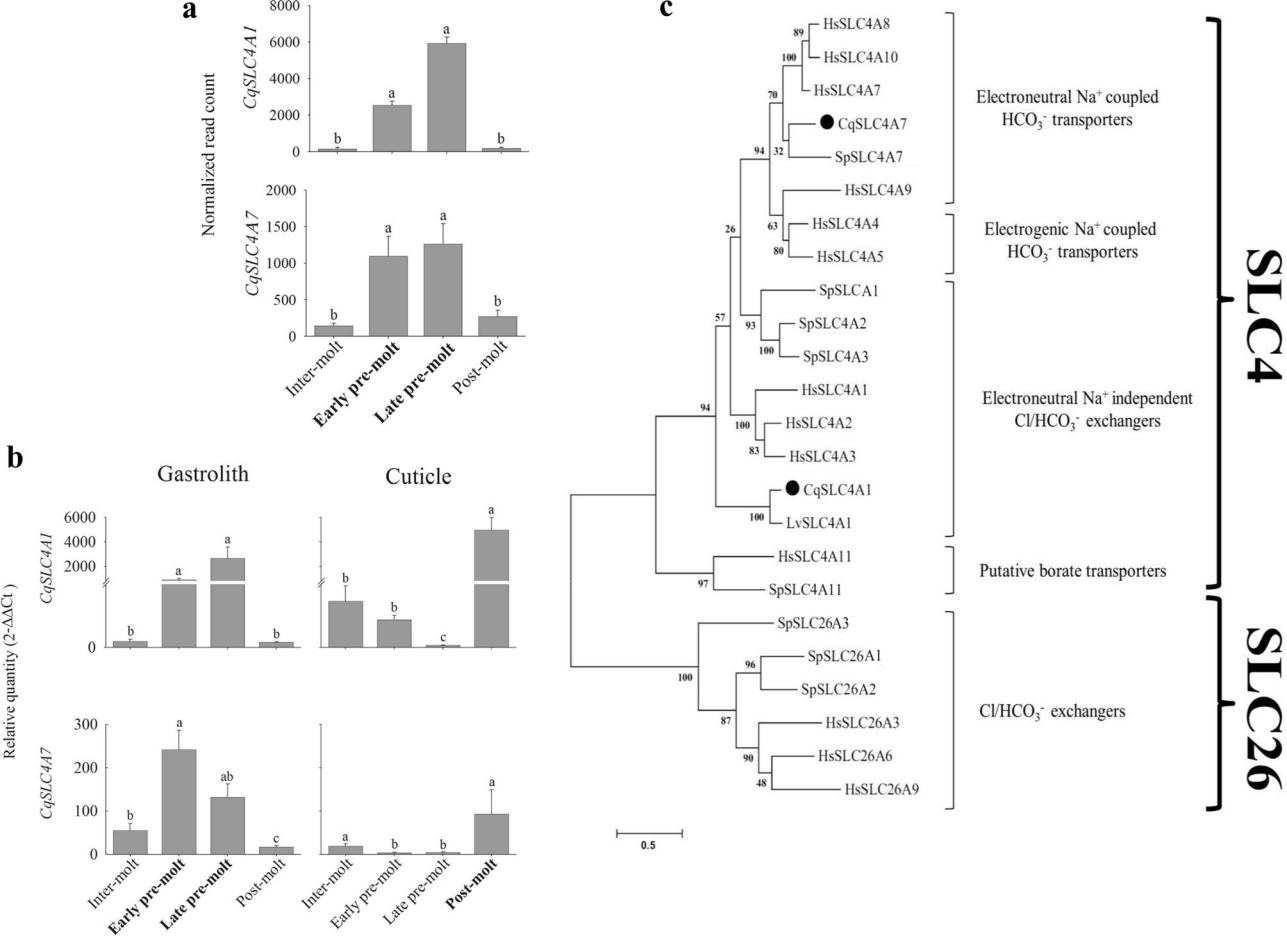
Gene expression levels of the newly found SLC4 transporters during the molt stages correlate with the mineralization patterns of distinct cuticular structures. (**a**) In-silico temporal expression of *CqSLC4A1* and *CqSLC4A7* found in the molt-related transcriptomic library. Data are means ± s.d.; inter-molt (n = 2), early pre-molt (n = 4), late pre-molt (n = 3) and post-molt (n = 3). Different letters represent groups that are statistically significantly different (p-value < 0.05). (**b**) Relative levels of *CqSLC4A1* (top) and *CqSLC4A7* (bottom) in the gastrolith-forming epithelium (left) and carapace cuticle-forming epithelium (right) at four molt stages, as determined by qPCR. Molt stages in which the bulk of mineralization occurs are shown in bold typeface for each cuticular structure. Data are means ± s.d., n = 5. Different letters represent groups that are significantly different (p < 0.05). (**c**) Maximum likelihood tree of SLC4 transport proteins from the decapod crustacean *C.* *quadricarinatus* (Cq) discovered in the present study and marked with a dot, the decapod crustacean *L.* *vannamei* (Lv), the bony coral *S.* *pistillata* (Sp), and *H. sapiens* (Hs). Bootstrap test (n = 1000) results are shown on each node junction.

In the first step of our effort to elucidate the bicarbonate transport mechanisms involved in crustacean biomineralization, we mined previously established large transcriptomic libraries^32^ for transport proteins involved in the biomineralization of cuticular structures of the crayfish *Cherax quadricarinatus.* In this crayfish species, calcium and bicarbonate ions are transferred at pre-molt from the old cuticle through the hemolymph to the gastroliths, transient amorphous calcium carbonate storage depots that are rich in phosphate^6,33^. We thus deemed it highly likely that such well-timed mineralization would be manifested in changes in the expression of genes involved in molecular transport mechanisms. Therefore, to reveal the mechanism/s of ion transport during the biomineralization processes occurring in the gastroliths of *C.* *quadricarinatus*, we employed an in silico mining binary patterning approach^32^ based on the molt-related transcriptomic library from the gastrolith-forming epithelium. In this approach, a binary pattern is assigned to the expression of genes during the four molt stages resulting in a four-digit code in which each position represents one molt stage. The first position in the code is for intermolt; the second, for early pre-molt; the third, for late pre-molt; and the fourth, for post-molt. Low expression is represented by 0, and high expression, by 1. We scanned the library for a 0110 binary pattern of expression (low expression at inter-molt and post-molt and high expression during the pre-molt stages)^32^ concomitant with the mineralization of the gastroliths and found four putative proteins involved in the transport of ions. These four candidate proteins included two putative bicarbonate transporters, which we designated CqSLC4A1 and CqSLC4A7 (accession numbers, MK890796 and MK890797, respectively) (Fig. 1a), and one pump predicted to support bicarbonate transport, i.e., the well-known sodium–potassium pump (Na^+^/K^+^-ATPase; accession number KR270438) (Fig. S1a). As opposed to mineralization of the gastroliths, which takes place at pre-molt, the outer cuticle of crustaceans is mineralized at post-molt, mainly by the absorption of calcium released from the gastroliths^2,6^. Using qPCR, we exploited the differences in the mineralization patterns of the gastroliths and cuticle to elucidate the role/s of CqSLC4A1 and CqSLC4A7 in the mineralization of these skeletal cuticular structures in accordance with the distinct mineralization timing of each structure. As expected, the two bicarbonate transporters exhibited inverse molt-related expression patterns in the gastrolith and cuticle: For both *CqSLC4A1* and *CqSLC4A7*, expression in the gastrolith was higher during the pre-molt stage, whereas in the cuticle the expression was higher at post-molt (Fig. 1b). During these molt stages in the latter cuticular structures calcium carbonate is being deposited^6,34^. Similar to the findings for the two putative bicarbonate transporters, qPCR assays of the predicted assisting pump, Na^+^/K^+^-ATPase, also showed inverse expression patterns in the gastroliths versus the cuticle (Fig. S1b). To obtain an indication of the generality of the molt-related pattern of expression of SLC4 proteins in crustaceans other than *C. quadricarinatus*, we compared the molt-related pattern of expression of *SLC4A1* in the cuticle of *C. quadricarinatus* to that of a distant crustacean relative, the decapod species *Litopenaeus vannamei*, the whiteleg shrimp. The expression patterns in the outer cuticles were indeed similar in the two decapod crustaceans, both exhibiting higher expression during post-molt (Fig. S2). In addition, the CqSLC4 proteins revealed by the library mining were widely conserved across taxonomic groups, spanning stony corals to humans (Fig. 1c).

**Figure 2:**
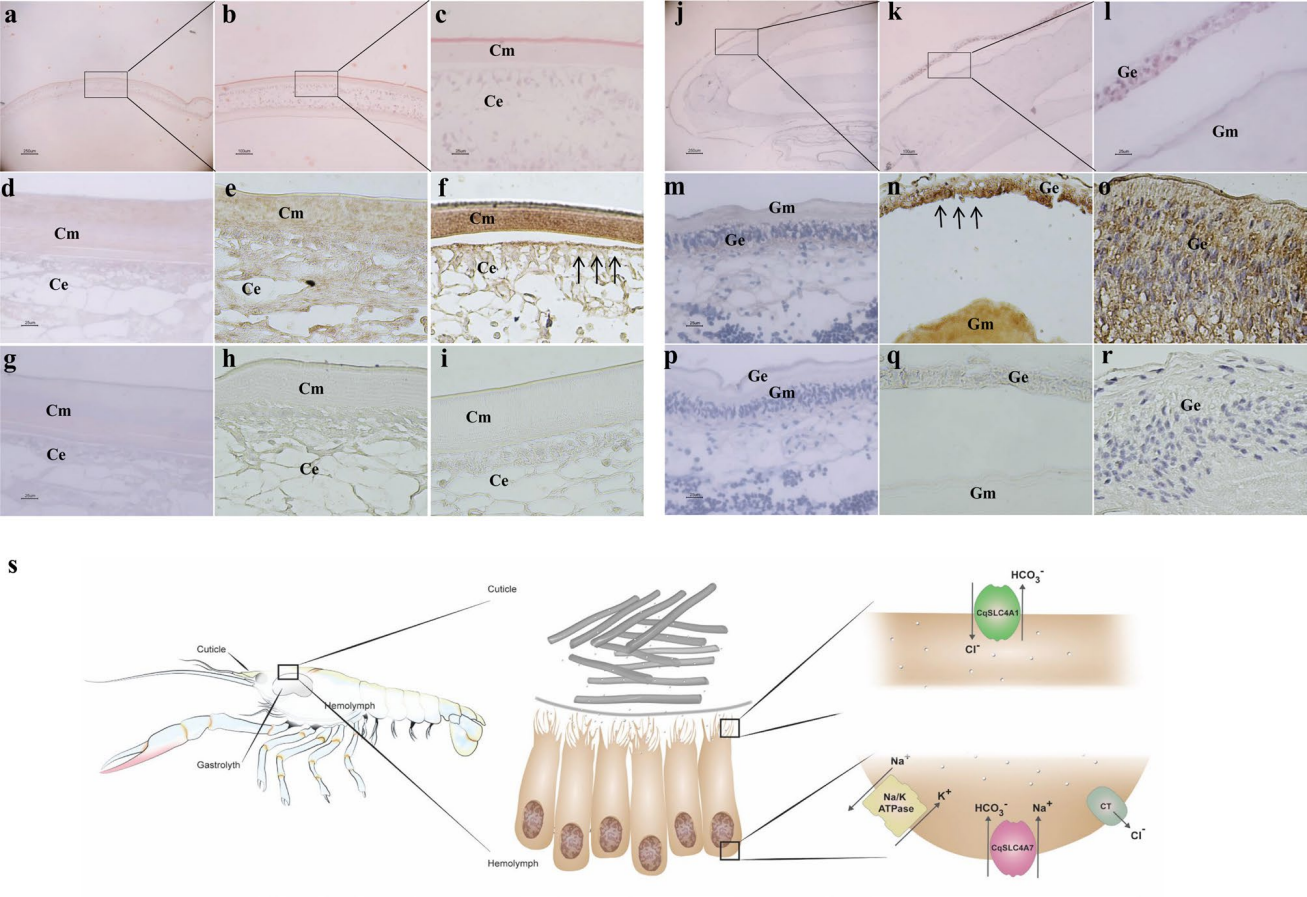
CqSLC4A1 is localized in the apical membrane of the forming epithelia of the cuticular structures of the crayfish during mineralization. (**a–c**) A representative carapace cuticle from a post-molt animal. H&E. (**a**) × 4. Black box and lines indicate the area magnified in (**b**) × 10. Black box and lines indicate the area magnified in (**c**) × 40. (**d–f**) Carapace cuticle incubated with anti-CqSLC4A1 from animals at different molt stages: (**d**) inter-molt, (**e**) late pre-molt, and (**f**) post-molt. (**g–i**) Carapace cuticle of control animals at different molt stages: (**g**) inter-molt, (**h**) late pre-molt, and (**i**) post-molt. (**j–l**) A representative gastrolith from a late pre-molt animal. H&E. (**j**) × 4. Black box and lines indicate the area magnified in (**k**) × 10. Black box and lines indicate the area magnified in (**l**) × 40. (**m–o**) Gastrolith incubated with anti-CqSLC4A1 from animals at different molt stages: (**m**) inter-molt, (**n**) late pre-molt, and (**o**) post-molt. (**p–q**) Gastrolith of control animals at different molt stages: (**p**) inter-molt, (**q**) late pre-molt, and (**r**) post-molt. Arrows indicate significant staining of anti-CqSLC4A1. *Cm* cuticular matrix, *Ce* cuticular epithelium, *Gm* gastrolith matrix and *Ge* gastrolith epithelium. (**s**) Schematic representations of the post-molt cuticular mineralization: Left—the molecular mechanism of bicarbonate transport in the forming epithelia of the cuticle and the gastrolith in a crayfish. Middle—forming epithelial cells underneath the cuticular extracellular chitinous scaffold. Right—putative locations of the different transporters in the epithelial cells and the predicted bicarbonate transport mechanisms involving CqSLC4A1 and CqSLC4A7 and transport-assisting mechanisms involving CqSLC12A2 and Na+/K+-ATPase.

Next, we undertook a molt-related immunohistochemical (IHC) investigation of CqSLC4A1 (Fig. 2, Supplementary Fig. S3) in the cuticle and gastroliths of *C. quadricarinatus*. Complementing the gene expression results, CqSLC4A1 was detected during post-molt in the cuticle (Fig. 2a–i) and during pre-molt in the gastrolith (Fig. 2j–r). Moreover, in both the gastroliths and the cuticle, CqSLC4A1 was found in the apical membrane of the forming epithelium facing the mineralizing chitinous extracellular matrix.

A schematic representation of the localization of CqSLC4A1 and CqSLC4A7 and the transport of bicarbonate ions in the forming epithelium cells is presented in Fig. 2s. CqSLC4A7, a symporter of sodium and bicarbonate, is depicted in the schematic representation at the basolateral membrane. We thus suggest a transport mechanism in which CqSLC4A7 is responsible for the inward flux of bicarbonate, maintaining the cellular homeostasis of bicarbonate and regulating cellular pH, while CqSLC4A1 is responsible for the outward transport of bicarbonate ions to the extracellular matrix into the mineralization site for both deposition of mineral and control over pH which is known to be controlled in the mineralization site of the crayfish gastroliths {Shechter, 2008 #49}. We also posit that a hypothetical chloride transporter and Na^+^/K^+^-ATPase are responsible for the equilibrium of cellular ionic homeostasis (Fig. 2s). As shown in mineralized tissues development, a wide variety of channels or transporters such as Na^+^/K^+^-ATPase and Cl^−^ transporters regulate or control multiple physiological and biological activities. These include pH regulation and calcium homeostasis, two processes important for the biomineralization and development^35^. Particularly in crustaceans, bicarbonate flux across the hypodermis of *C. sapidus* was found to be associated with high levels of carbonic anhydrase (CA) activity during the time of maximal resorption during premolt which then decrease markedly just before the molt^36^. CA facilitate the diffusion of HCO_3_^−^ across phospholipid membranes^37^. During cuticular resorption, the dissolution of CaCO_3_ liberates HCO_3_^−^ while membrane-bound CA can catalyze the conversion of H^+^ + HCO_3_^−^ to CO_2_ + H_2_O. Carbon dioxide can diffuse across unstirred layers and lipid membranes more easily and quickly than HCO_3_^−^. Inside the epithelial cells CA can catalyze the reverse reaction. The movement of HCO_3_^−^ across the basolateral membrane into the hemolymph is likely accomplished by a Cl^−^/HCO_3_^−^ exchanger (CHE) and some form of active HCO_3_^−^ transport^38^ as suggested in our hypothetical model (Fig. 2s).

### Elucidating the role of the CqSLC4 family

**Figure 3:**
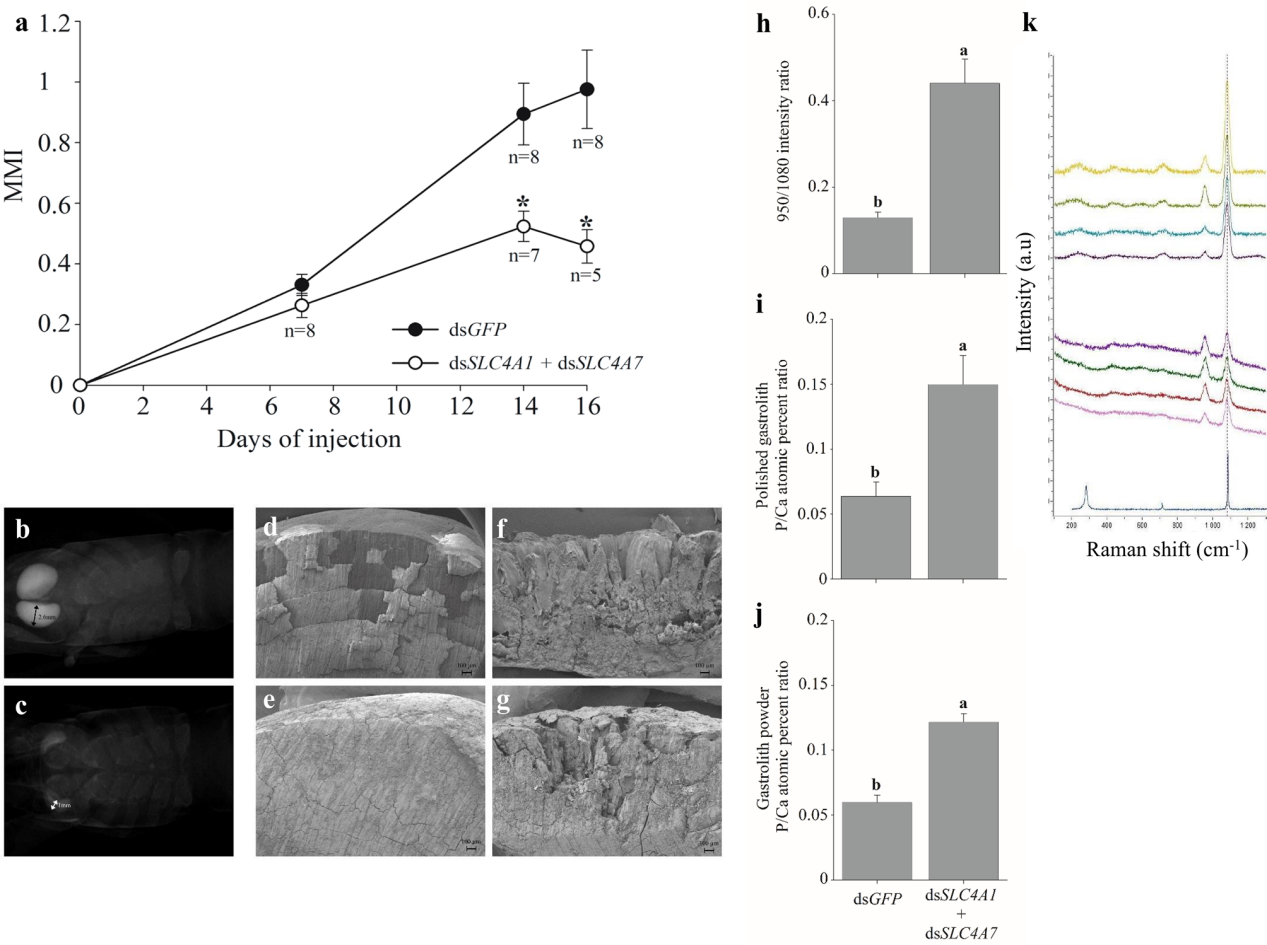
Effects on gastrolith mineralization following loss-of-function of CqSLC4 through RNAi. (**a**) The mineralization of the gastroliths is shown in terms of the molt mineralization index (MMI) of animals in the control group injected with ds*GFP* (n = 8) and of animals in the treatment group injected with ds*SLC4A1* + ds*SLC4A7* (n = 8, day 7), (n = 7, day 14) and (n = 5, day 16). Data are means ± s.d. Asterisk (*) represents statistically significant difference (p-value < 0.05) between the ds*GFP* and ds*SLC4A1* + ds*SLC4A7* at the same time point. (**b,c**) Representative X-ray images of gastroliths of: (**b**) ds*GFP*-injected control crayfish and (**c**) silenced crayfish injected with ds*SLC4A1* + ds*SLC4A7*. (**d–g**) Representative SEM images of gastrolith cross sections of: (**d,e**) control crayfishes and (**f,g**) silenced crayfishes. Phosphate to calcium ratio in the gastroliths of the treatment group injected with ds*SLC4A1* + ds*SLC4A7* versus the control group injected with ds*GFP* is shown using various techniques. (**h**) Ratio of the intensities of the Raman peaks at 950 and 1080 cm-1 for control (n = 8) versus treatment animals (n = 8). (**i**) Ratio between the atomic percentages of phosphate and calcium in a polished gastrolith (cross section) of a control animal (n = 8) versus a silenced animal (n = 8). (**j**) EDXS-derived ratio of the atomic percentages of phosphate and calcium of gastrolith powder from control (n = 8) versus silenced animals (n = 8). For all graphs different letters represent groups that are significantly different (p < 0.05); bars represent standard error. (**k**) Representative Raman spectrum with peaks at 950 and 1080 cm^−1^, indicating the presence of amorphous calcium phosphate and amorphous calcium carbonate, respectively. The four traces at the top of the figure are those for a gastrolith of a representative control animal; the four traces in the middle of the figure relate to a gastrolith of a representative treatment animal; and the bottom trace is that for pure calcite as a reference.

To determine the role of the newly found CqSLC4 proteins in the formation of the skeletal cuticular structures, RNAi-based loss-of-function experiments were performed (Fig. S4). The first loss-of-function experiment was performed in crayfish that had been induced to molt by daily injections of ecdysone, which is the hormone responsible for molting in crustaceans^39^ (Fig. S5). This silencing experiment was performed at pre-molt with the aim to target the mineralization of the gastroliths. This experimental design was chosen because gastrolith mineralization is easily monitored using a known proxy for molt stages, the molt mineralization index (MMI), which provides a measure of the relative gastrolith size by using a non-invasive X-ray technique^6^. We detected a profound impact of silencing on the mineralization of the gastroliths in that the MMI of silenced animals injected with a 50:50 mixture of ds*CqSLC4A1* + ds*CqSLC4A7* and ecdysone*—*as compared with control animals injected with ds green fluorescent protein (ds*GFP*) and ecdysone—was significantly reduced, starting from day 14 of the injections (Fig. 3a). In addition, the MMI of non-ecdysone induced animal was unchanged as compared to induced animals (Fig S5). X-rays of the gastroliths of the silenced animals showed a significant reduction in size and extent of mineralization vs the gastroliths of control animals along the entire silencing experiment (Fig. 3b,c). SEM images of gastroliths from the silenced group vs the control group (Fig. 3d–g) further demonstrated the effect of silencing in that they showed reduced and deficient mineralization and even the presence of bare chitin lamellas in the treated gastroliths. It is important to note that our silencing experiments targeted the gastroliths mineralization at pre-molt since silenced crayfish did not survive molt to enable testing for differences in post-molt cuticular mineralization.

Phosphate, being the third most abundant ion in the gastroliths, plays an important role in the stabilization of the amorphous calcium carbonate in these organs^33^. We expected that silencing the CqSLC4 transporters family would reduce the bicarbonate transport and lead to an increase in the Phosphate:Calcium ratio in the gastroliths.

Raman spectra of gastroliths exhibit two characteristic vibrational modes at 950 and 1080 cm^−1^ corresponding to amorphous calcium phosphate (ACP) and amorphous calcium carbonate (ACC), respectively. As expected, silenced gastroliths showed a significant increase in the ACP:ACC ratio of ≈ 60% (Fig. 3h,k). EDXS analysis of polished and powdered gastroliths (Fig. 3i,j) also shows that silenced gastroliths exhibit significantly higher atomic percentages of phosphate than non-silenced samples.

The above monitoring of the mineral composition by Raman and EDXS following loss of function of CqSLC4 transporters provides strong evidence for the role of these transporters in gastrolith mineralization.

An interesting question arising from these results is what is the cause of the increase in phosphate relative to calcium due to silencing? Such an increase is probably due to a reduction in the deposition of calcium. We hypothesize that since to deposit calcium, carbonate is necessary as well, any interruption in carbonate supply results in lesser deposition of calcium while the mechanisms of phosphate transport is unharmed. Our model for the deposition of various calcium minerals in the cuticular structures of *C.* *quadricarinatus* suggests that the newly discovered putative transporters CqSLC4s provide carbonate for the mineralization site of the cuticular structures which is than coupled to calcium and deposited in either amorphous or crystalline phases. In addition, as known in crustaceans, phosphate is being deposited^40,41^ and might be using a different mechanism of transport. The reason for which calcium phosphate is not deposited in the mineralization site following our loss of function experiment is probably due to the fact that phosphate accounts for only 4–18% in the gastroliths of *C. quadricarinatus*^42^ therefore even after silencing it is not the most abundant ion, and calcium carbonate is still being deposited as can be seen in our Raman results (Fig. 3h,k).

**Figure 4:**
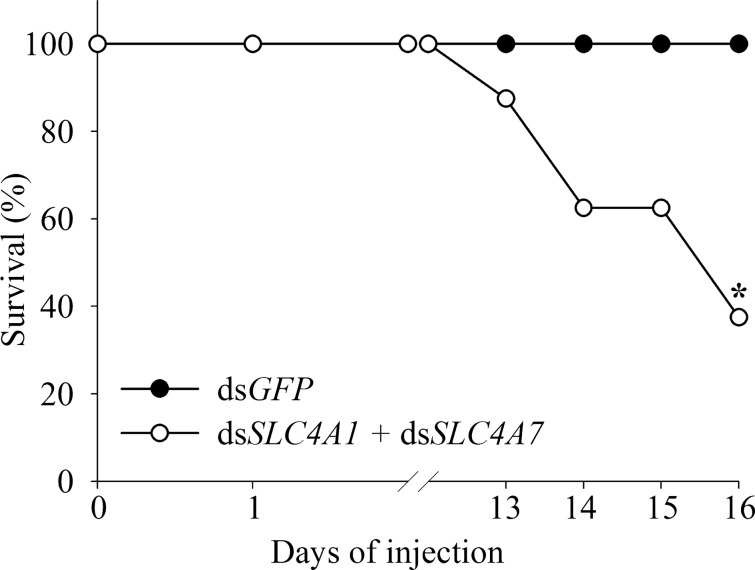
Effects of CqSLC4 silencing on survival during the molt cycle of *C.* *quadricarinatus*. Graph showing survival rates during pre-molt. The control group was injected with ds*GFP* (n = 8); the treatment groups were injected with ds*SLC4A1* + ds*SLC4A7* (n = 8). All groups were given ecdysone injections to induce molting. Asterisk indicates significant difference in survival.

An additional finding of importance was a significant decrease in the survival of the silenced group as compared with the control group (Fig. 4); this finding seems to be related to molt, since a similar decrease in survival was not found in a second silencing experiment in which the crayfish were not induced to molt (Fig. S6). We thus conclude that the gastrolith mineralization-and-development phenotype may be attributed to a disruption in the supply of bicarbonate ions for mineralization. Our RNAi silencing results therefore suggest that the role of the putative CqSLC4 transporters is related to the supply of bicarbonate ions for hardening of the cuticular matrix in the crustacean cuticle. Of particular importance, these results constitute the first evidence for the involvement of SLC4 proteins in the supply of bicarbonate ions to a biomineralization site. Previous studies on the involvement of SLC4 members in biomineralization have relied solely on indirect evidence, such as the pattern of expression of SLC4 genes^23,25,27^ or the localization of SLC4 proteins^27^, implying that the role of these proteins might have been related to other functions of the tissue. It is important to note that further study will be needed to demonstrate the direct function of CqSLC4’s in the transport of bicarbonate. Moreover, molecular research regarding the supply of bicarbonate is hard to resolve, since inorganic carbon can diffuse through the cell membrane and its transformation to bicarbonate is pH dependent. As such, previous models of calcium carbonate biomineralization in other organisms have raised questions regarding the nature of carbonate transport to the site of mineralization^34^.

### CqSLC4 coupling molt and mineralization

**Figure 5:**
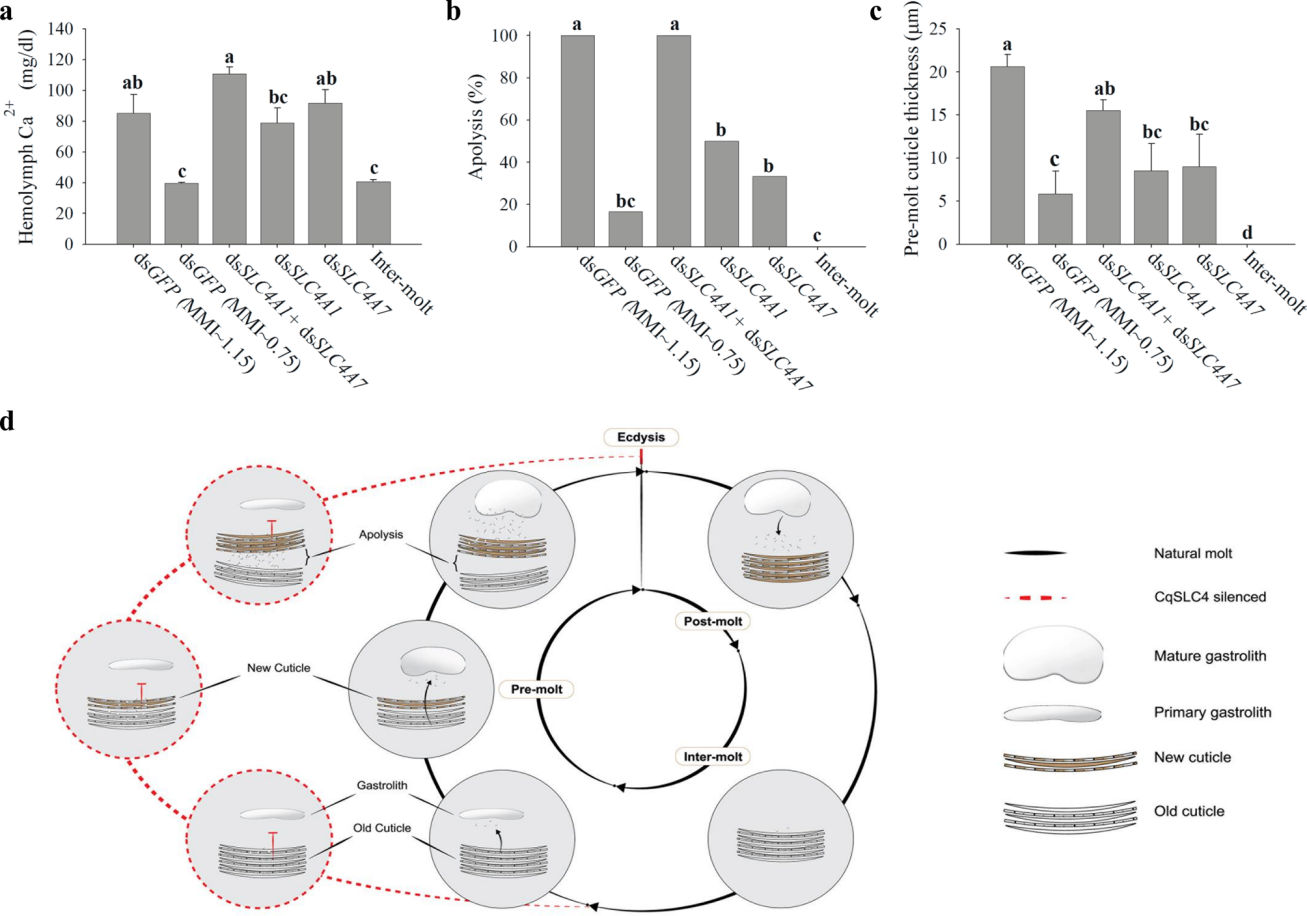
Temporal silencing of SLC4 transporters during the molt cycle leads to uncoupling of molt and mineralization. (**a**–**c**) The molt-related physiological parameters, Ca2+ concentration in the hemolymph, apolysis, and pre-molt cuticle thickness, are shown for three treatment groups silenced with dsSLC4A1 + dsSLC4A7, dsSLC4A1, or dsSLC4A7 and harvested at MMI ≈ 0.75, a negative control group harvested at inter-molt (MMI = 0) versus two control groups injected with dsGFP and harvested at either MMI ≈ 0.75 or prior to molt at MMI ≈ 1.15. (**d**) Schematic representation of the coupling between the mineralization of cuticular structures of *C.* *quadricarinatus* and molt cycle events. Molt cycle events occurring in the two main cuticular structures of *C.* *quadricarinatus*, the cuticle and the gastrolith, namely, the mineralization of the gastroliths at pre-molt, the demineralization of the old cuticle at pre-molt, the transfer of calcium from the old cuticle to the hemolymph at pre-molt, the formation of a new non-mineralized cuticle underneath the old cuticle at pre-molt, apolysis and separation of the newly forming cuticle from the old cuticle at late pre-molt, demineralization of the gastroliths at post-molt, mineralization of the newly formed cuticle at post-molt, and formation of a mature cuticle at inter-molt. The right side of the figure shows uncoupling of gastrolith mineralization from the different molt cycle events during pre-molt due to silencing of CqSLC4A1 and SLC4A7 during pre-molt.

The power of using a crustacean model in the study of biomineralization—and the reason for our success in resolving the mechanism of bicarbonate transport—lies in the tight coupling of the molt cycle to mineralization and demineralization of the crustacean cuticular structure^43^. To demonstrate the role of the SLC4 members in this important coupling between the molt cycle events and mineralization, we performed a third silencing experiment, in which crayfish were also induced to molt (by administering daily ecdysone injections). The main aim of the third silencing experiment was to monitor three indicators of ecdysis (the act of shedding the old cuticle): (i) the concentration of calcium in the hemolymph, (ii) the occurrence of apolysis (which is the process by which the new cuticle separates from the old cuticle prior to molt), and (iii) the thickness of the newly forming cuticle. In this experiment, the treatment groups were subjected to pre-molt silencing of the putative *CqSLC4A1* and *CqSLC47* transporters, each separately, or in a 50:50 mixture, since silencing a single transporter was found to affect some of the molt cycle indicators but not at the same intensity as the combined silencing. For this experiment, there were two control groups; both were injected with ds*GFP* (Fig. 5), but one group was collected at mid pre-molt (MMI ≈ 0.75) and the other, close to ecdysis at late pre-molt (MMI ≈ 1.15). The three silenced treatment groups were all harvested at mid-premolt (MMI ≈ 0.75). The three indicators of ecdysis in the treatment groups were found to be similar to those in the group of control animals harvested at late pre-molt just before the ecdysis event (MMI ≈ 1.15) and significantly different from those in the control animals harvested at a similar MMI, indicative of mid pre-molt (MMI ≈ 0.75) (Fig. 5a–c). These results suggest that in silenced crayfish the molt cycle events progressed at a normal pace all the way up to the verge of ecdysis, while the mineralization of the gastrolith did not progress accordingly*,* indicating an unnatural uncoupling of gastrolith mineralization from the molt cycle (described schematically in Fig. 5d). This uncoupling might have been the cause of the decline in survival of the silenced crayfish described above due to disturbance in the transport of calcium and bicarbonate ions to the gastroliths^2,6^ and the consequent perturbation of ionic homeostasis. These results demonstrate the impact of the putative CqSLC4 transporters on crayfish physiology at the precise time of mineralization during molt and formation of skeletal cuticular structures. Such a tight coupling between molt and mineralization contributes to the extraordinary fitness of crustaceans in terms of their ability to mineralize and demineralize cuticular structures with extreme accuracy and efficiency. Such an efficiency is especially necessary since crayfish inhabit a freshwater low calcium environment. We therefore suggest that SLC4 transporters play a central role in the coupling between molt and mineralization of crustaceans (Fig. 5d). The discovery of a cyclic control of SLC4 transporters in crustaceans could be seen in light of what has been found in mammalian ameloblasts and corals^21,27^. However, future studies should reveal the mechanistic switch which occurs in these epithelia. The involvement of SLC4 transporters in controlling the mineralization of skeletal cuticular structures of crustaceans during the molt cycle represents a missing link in the evolution of ion transport mechanism in biomineralizing organisms. The present study adds a layer of knowledge regarding this family of transporters in that it demonstrates the significance of their role in biomineralization in addition to their well-known role in pH regulation^28,44^. Therefore, our results provide significant insights regarding the fascinating process of calcium carbonate biomineralization.

## Materials and methods

### Animals and molt induction

*Cherax quadricarinatus* crayfish were grown in and collected from artificial ponds at Ben-Gurion University of the Negev (BGU), Beer-Sheva, Israel, as previously described^45^. For all the molt induction experiments, inter-molt crayfish were held in individual cages and endocrinologically induced to enter pre-molt through daily α-ecdysone injections, as previously described^32^. Progression of the molt cycle was monitored daily by measuring the gastrolith MMI, which is known to correlate with molt stages and hormonal titers^6^. MMI values for the molt stages were: inter-molt, 0; early pre-molt, 0.02–0.04; mid pre-molt, 0.05–0.08; and late pre-molt 0.1–0.2. Post-molt animals were harvested on the day following ecdysis. For all dissection procedures, crayfish were placed on ice for 10–15 min until they became anesthetized.

### Mining for genes related to ion-transport in cuticular structures

Mining for candidate transport proteins involved in the formation of the cuticular structures of *C.* *quadricarinatus* was conducted on the basis of the molt-related transcriptomic library described previously^32^. Detection of ion transport proteins genes was performed using the binary-patterning mining approach described earlier^32^. With this approach, transcripts showing a pattern of expression which is concomitant with gastrolith mineralization meaning high expression at pre-molt as compared with inter-molt and post-molt, (binary pattern 0110), were grouped and regarded as potential candidate transcripts. The filtered candidate transcripts were then computationally translated to proteins using the translate tool on the ExPASy Proteomics Server^46^ (http://ca.expasy.org/tools/dna.html “\h). The identification and annotation of ion-transport proteins in the filtered transcripts were performed using SMART^47^. Differences in the timing of protein expression in the cuticular structures of *C.* *quadricarinatus* were tested using qPCR in the gastrolith-forming epithelium and in the cuticle; candidate transporters showing inverse patterns of expression (for pre-molt and post-molt) were regarded as putative mineralization-related transport proteins in the cuticular structures of *C.* *quadricarinatus*. To perform the qPCR, total RNA was isolated from the gastrolith and carapace cuticle-forming epithelium from male animals at four main molt stages: inter-molt, early pre-molt, late pre-molt and post-molt (n = 5 for each molt stage). RNA isolation and cDNA preparation were performed as described in ref.^48^*.* In addition, RNA was extracted from the muscle tissue of one inter-molt animal for normalization. Relative quantification of transcript levels was performed using Roche Diagnostics FastStart Universal Probe Master Mix (Basel, Switzerland) and Roche Universal Probe Library probes. The following primers and probes were used: for *CqSLC4A1*, q*cqSLC4A1*F and q*cqSLC4A1*R, Probe #38; for *CqSLC4A7*, q*cqSLC4A7*F and q*cqSLC4A7*R, Probe #144; for *Na*^+^*/K*^+^*-ATPase subunit α*, q*cqNaK*F and q*cqNaK*R, Probe #154. *C.* *quadricarinatus 18S*, which served as a normalizing gene, was also quantified by means of real-time RT PCR using the primers, q*cq18S* F and q*cq18S* R, Probe #22. All primer sequences are shown in Table S1. Reactions were performed with the ABI Prism7300 Sequence Detection System, Applied Biosystems (Foster City, CA). Statistical analyses for relative expression levels between the molt stages were performed using the non-parametric Kruskal–Wallis rank sum test, followed by multiple pair-wise comparisons using the Wilcoxon rank sum test; *p* < 0.05 was considered statistically significant. In addition, to test for the possible involvement of SLC4A1 in the mineralization of the cuticular structures of other crustaceans, we determined the expression pattern of SLC4A1 from the white leg shrimp *L.* *vannamei* (accession number XP_027221056) in a previously published molt-related transcriptomic library^49^.

### Phylogenetic analysis

For phylogenetic analysis, protein sequences of SLC4 bicarbonate transporters and members of another known bicarbonate transporter family SLC26 from *Homo sapiens*^28^ and the stony coral *Stylophora pistillata*^27^ were retrieved from the NIBN gene bank. These sequences plus the two SLC4 protein members that are the focus of this study and that were found in *C.* *quadricarinatus* and SLC4A1 from *L.* *vannamei* were utilized to build a maximum likelihood tree with MEGA6 software5.1, using the Poisson model, uniform rates among sites, complete deletion of gaps/missing data and a very strong branch swipe filter coupled to a bootstrap test set at 10 × 10^3^ repetitions.

### Extraction and western blot of membrane proteins

Membrane proteins were extracted from the cuticle-forming epithelium of post-molt animals and were further purified on a lectin column packed with agarose-bound concanavalin A (Vector Lab., USA). The elution of the lectin column was than separated in two gels by SDS-PAGE using MOPS running buffer (4–20% GenScript gels). For western blot, one gel was transferred to a nitrocellulose membrane. Following blocking of the membrane with 3% skim milk in Tris-buffered saline (TBS) containing 0.1% Tween-20 (TBST), the membrane was incubated overnight (4 °C) with anti-CqSLC4A, at a dilution of 1:250 (v/v). After washing 3 times with TBST, the membrane was incubated with horseradish peroxidase (HRP)-conjugated goat anti rabbit immunoglobulin G secondary antibody at a dilution of 1: 10,000, v/v (Santa Cruz, USA). Antibody binding was detected with an EZ-ECL chemiluminescence detection kit (Biological Industries, Beit Haemek, Israel). Based on the immunoblot detected bands, candidate CqSLC4 proteins were visualized with Coomassie Blue (CBB) in the stained gel.

### Histology and immunohistochemistry

Carapace cuticle and gastroliths (including the forming epithelium) of animals from three molt stages (inter-molt, late pre-molt and post-molt) were decalcified in a histological decalcifying agent (Calci-Clear Rapid; National Diagnostics, Atlanta, GA) and fixed with paraformaldehyde (4%). Samples were then dehydrated, embedded in paraffin, and sectioned as previously described^50^. Sections (5 µm) were stained with hematoxylin & eosin (H&E) and observed under a light microscope. For IHC, polyclonal antibodies against CqSLC4A1 (anti-CqSLC4A1) were developed in a rabbit immunized with two peptides from the CqSLC4A1 protein sequences, -SDKGFHEVAYRAHSRGC- and -PSEWNNKDLVPIDDIRAKC- (Adar Biotech, Rehovot, Israel). The reason why we chose CqSLC4A1 for IHC is that it was found to be the most expressed protein in our previous results. For enhanced specificity, the immunosera were passed through a peptide affinity column. Slides were then deparaffinized and washed with TBS. For antigen retrieval, slides were incubated at 95 °C for 30 min in citrate buffer (0.5 M, pH 6). For blocking non-specific binding of antibodies, slides were then incubated with 2% normal goat serum and 2% fish skin gelatin in TBS (blocking solution) for 2 h at room temperature. Thereafter, slides were incubated at 4 °C overnight with the primary antibody anti-CqSLC4A1diluted in the blocking solution at a dilution of 1:100 (v/v). Control group slides were incubated with blocking solution alone. To quench endogenous peroxidase activity, slides were incubated with 3% H_2_O_2_ in TBS for 15 min. Finally, slides were washed in TBS and incubated with HiDef DetectionTM HRP polymer system (Cell Marque, Rocklin, CA) for 10 min. Then, the bound antibody was visualized by incubating the slides in 3,3′-diaminobenzidine (DAB) chromogen (Cell Marque, Rocklin, CA). Slides were then counter-stained with hematoxylin, mounted and examined under a microscope.

### dsRNA production and silencing efficiency

Loss-of-function experiments using RNAi were conducted for *CqSLC4A1* and *CqSLC4A7*. To synthesize dsRNA, two PCR products were generated using a T7 promoter anchor (T7P) attached to one of the two primers used to amplify each product. The primers used for generating the template for *CqSLC4A1* and *CqSLC4A7* sense-strand RNA synthesis were: as the forward primer ds*cqSLC4A1*F + T7 and ds*cqSLC4A7*F + T7, respectively, and as the reverse primer ds*cqSLC4A1*R and ds*cqSLC4A7*R, respectively. For *CqSLC4A1* and *CqSLC4A7* anti-sense strand RNA synthesis, the forward primers were ds*cqSLC4A1*F and ds*cqSLC4A7*F, respectively, and the reverse primers were ds*cqSLC4A1*R + T7 and ds*cqSLC4A7*R + T7, respectively. All primer sequences are shown in Table S1. PCR amplicons were separated and visualized, followed by dsRNA preparation, hybridization, quantification and maintenance, as described previously^51^. To test for silencing efficiency, inter-molt *C.* *quadricarinatus* males (10–15 g) were induced to molt. Animals were divided into three groups, a treatment group injected with dsRNA of *CqSLC4A1* (n = 6), a treatment group injected with dsRNA of *CqSLC4A7* (n = 6), and a control group injected with an exogenous dsRNA of *GFP* (n = 6). The concentration of dsRNA was 5 µg per g body weight. This concentration of dsRNA was utilized since it is known to induce silencing in *C.* *quadricarinatus*^52^*.* The injections of dsRNA began at an MMI of ≈ 0.07 and were given daily over a period of 5 days. On the sixth day, the animals were dissected, and mRNA from the gastrolith-forming epithelium was extracted and cDNA was prepared, as described above. The relative expression of *CqSLC4* encoding genes in the gastrolith-forming epithelium of the different groups was evaluated using qPCR, and statistical analyses were performed as described above.

### Phenotypic effects following RNAi

To test for phenotypic effects in gastrolith formation, three loss-of-function experiments were conducted; the two main experiments were conducted in parallel to molt induction. In the first experiment aimed to understand the influence of silencing on gastrolith mineralization, inter-molt *C. quadricarinatus* males (5–10 g) were induced to molt and divided to two groups (n = 8 for all groups), a treatment group injected with a 50:50 mixture of dsRNA of *CqSLC4A1* (ds*SLC4A1*) and dsRNA of *CqSLC4A7* (ds*SLC4A7*) and a control group injected with dsRNA of *GFP* (ds*GFP*). The dsRNA was given in a *SLC4A1–SLC4A7* mixture to prevent compensation mechanisms and to enhance the effect of silencing. The dsRNA administration protocol was 5 µg dsRNA/g body weight injected every 2 days over a period of 16 days (by that time the survival of the silenced group was significantly reduced, and injections were therefore stopped). The MMI of the animals was measured as described in ref.^6^ at three time points: after 7 days, after 14 days, and on the 16th day. At the end of the experiment, the animals were anesthetized, as described above, and the newly formed gastroliths were dissected out. Differences in MMI between control and silenced group values were tested using the Kruskal–Wallis rank sum test, followed by multiple pair-wise comparisons using the Wilcoxon rank sum test. Differences in survival between the control and silenced groups were tested using the chi square test of fitness. In all statistical analyses, p < 0.05 was considered statistically significant.

The second silencing experiment was conducted without molt induction to determine the effect of silencing on survival during inter-molt. For that purpose, inter-molt male *C.* *quadricarinatus* (5–10 g) animals were used. dsRNA injections were given as described above over a period of 30 days, with no injections of ecdysone. In this experiment, the animals were divided to four groups, a treatment group injected with ds*SLC4A1* + ds*SLC4A7,* a treatment group injected with ds*SLC4A1*, a treatment group injected with ds*SLC4A7*, and a control group injected with ds*GFP* (n = 7 for all groups).

The third experiment was aimed at understanding the molt-related physiological changes at late pre-molt prior to ecdysis induced by the silencing of the CqSLC4 transporter proteins. The experiment was conducted on early pre-molt male *C.* *quadricarinatus* (5–10 g) animals, who were induced to advance in the molt cycle as described earlier. Preliminary experiments had shown that survival of pre-molt animals silenced with ds*SLC4A1* + ds*SLC4A7* can be achieved until a stage in the molt cycle when the MMI is ≈ 0.75; the experiment was therefore planned in accordance with these preliminary survival results. dsRNA injections and molt induction were performed as described above. To study indicators of the progression of the molt cycle and their coupling to/uncoupling from mineralization of the gastroliths, animals were divided to five groups (n = 6 for all groups)—three silenced and two control. One silenced group was injected with ds*SLC4A1* + ds*SLC4A7*, one with ds*SLC4A1*, and one with ds*SLC4A7*. Both control groups were injected with ds*GFP*; one control group was harvested at mid pre-molt, i.e., MMI ≈ 0.75, and the other, at late pre-molt close to ecdysis at MMI ≈ 1.15. The experiment was stopped when an MMI ≈ 0.75 was reached in the treatment groups. At the end of the experiment, animals were anesthetized, as described above, the newly formed cuticle was dissected (if present), and cuticle thickness was measured as described earlier^48^. The occurrence of apolysis was monitored, and hemolymph serum was extracted and tested for calcium concentration using a Vetscan VS2 (Abaxis Inc., Union City, CA). In addition, hemolymph serum was extracted from six inter-molt animals to determine the basal concentration of calcium in the serum.

Silencing experiments aiming at understanding the role of the newly found CqSLC4’s in cuticular mineralization were not performed. This is due to the fact that silencing was found to be lethal prior to the mineralization of the cuticle at post-molt.

### SEM and EDXS

Gastroliths and newly formed cuticle dissected out in the various silencing experiments were cleaned and air dried. Samples were either characterized with a JEOL JSM-7400f electron microscope at the Ilse Katz Institute for Nanoscale Science & Technology (IKI), BGU, Beer-Sheva, Israel or at the Center for Nanoscience and Nanotechnology, The Hebrew University of Jerusalem, using an FEI Quanta 200 ESEM in low-vacuum mode without preliminary treatment. For SEM images, polished gastroliths and manually fractured cuticles were gold coated for 6 s and viewed under an electron microscope at the IKI. For EDXS and determination of carbonate and phosphate contents, gastroliths from the first silencing experiment were either gently polished in dry conditions in order to avoid differential dissolution effects and viewed at the IKI under an electron microscope with an EDXS detector (Kevex; Thermo Scientific, Scotts Valley, CA; the acceleration voltage was set at 10 kV^53,54^) or ground to a powder and viewed under an electron microscope at the Center for Nanoscience and Nanotechnology (acceleration voltage set at 20 kV).

### Raman spectroscopy

Gastroliths extracted from animals in the first silencing experiment aimed to understand the influence of silencing on gastrolith mineralization were cleaned, air dried and polished and subjected to Raman spectroscopy. The Raman system comprised a Horiba LabRam HR evolution micro-Raman system, equipped with a Synapse Open Electrode CCD detector air-cooled to − 60 °C. The excitation source was a 633 nm laser with power on the sample of 10 mW. The laser was focused with a × 50 objective to a spot of about 2 µm. The measurements were taken with a 600 lines mm^−1^ grating and a 100 µm confocal microscope hole. The exposure time was 30 s. For each sample, four random points were tested for the intensities at 950 and 1080 cm^−1^, indicating the presence of amorphous calcium phosphate (ACP) and amorphous calcium carbonate (ACC) respectively.

## Supplementary Information

Supplementary Information.

## Data Availability

All data is available in the main text or the supplementary materials. Additional data available from authors upon request.

## References

[1] Lowenstam HA, Weiner S (1989). On Biomineralization.

[2] Roer R, Dillaman R (1984). The structure and calcification of the crustacean cuticle. Am. Zool..

[3] Edgecombe GD, Legg DA (2014). Origins and early evolution of arthropods. Palaeontology.

[4] Bentov S, Abehsera S, Sagi A (2016). Extracellular Composite Matrices in Arthropods.

[5] Bentov S, Aflalo ED, Tynyakov J, Glazer L, Sagi A (2016). Calcium phosphate mineralization is widely applied in crustacean mandibles. Sci. Rep..

[6] Shechter A (2008). Reciprocal changes in calcification of the gastrolith and cuticle during the molt cycle of the red claw crayfish <i>Cherax quadricarinatus</i>. Biol. Bull..

[7] Welinder BS (1974). The crustacean cuticle—I. Studies on the composition of the cuticle. Comp. Biochem. Physiol. A Physiol..

[8] Roer R, Abehsera S, Sagi A (2015). Exoskeletons across the Pancrustacea: Comparative morphology, physiology, biochemistry and genetics. Integr. Comp. Biol..

[9] Skinner DM (1962). The structure and metabolism of a crustacean integumentary tissue during a molt cycle. Biol. Bull..

[10] Marsh ME (2003). Regulation of CaCO3 formation in coccolithophores. Comp. Biochem. Physiol. B Biochem. Mol. Biol..

[11] Uriz MJ (2006). Mineral skeletogenesis in sponges. Can. J. Zool..

[12] Von Euw S (2017). Biological control of aragonite formation in stony corals. Science.

[13] Arends J, Jongebloed WL (1981). Apatite single crystals. Formation, dissolution and influence of CO32− ions. Recl. Trav. Chim. Pays Bas.

[14] Rey C, Collins B, Goehl T, Dickson IR, Glimcher MJ (1989). The carbonate environment in bone mineral: A resolution-enhanced Fourier Transform Infrared Spectroscopy Study. Calcif. Tissue Int..

[15] Bushinsky DA, Krieger NS, Geisser DI, Grossman EB, Coe FL (1983). Effects of pH on bone calcium and proton fluxes in vitro. Am. J. Physiol..

[16] Bushinsky DA, Sessler NE (1992). Critical role of bicarbonate in calcium release from bone. Am. J. Physiol..

[17] Bushinsky DA (2003). Chronic acidosis-induced alteration in bone bicarbonate and phosphate. Am. J. Physiol. Renal Physiol..

[18] Lacruz RS, Nanci A, Kurtz I, Wright JT, Paine ML (2010). Regulation of pH during amelogenesis. Calcif. Tissue Int..

[19] Lacruz RS (2012). Requirements for ion and solute transport, and pH regulation during enamel maturation. J. Cell. Physiol..

[20] Hu MY (2018). A SLC4 family bicarbonate transporter is critical for intracellular pH regulation and biomineralization in sea urchin embryos. Elife.

[21] Jalali R (2014). NBCe1 (SLC4A4) a potential pH regulator in enamel organ cells during enamel development in the mouse. Cell Tissue Res..

[22] Jansen ID (2009). Ae2a, b-Deficient mice exhibit osteopetrosis of long bones but not of calvaria. FASEB J..

[23] Mackinder L (2011). Expression of biomineralization-related ion transport genes in *Emiliania huxleyi*. Environ. Microbiol..

[24] Paine ML (2008). Role of NBCe1 and AE2 in secretory ameloblasts. J. Dent. Res..

[25] Voigt O (2017). Spicule formation in calcareous sponges: Coordinated expression of biomineralization genes and spicule-type specific genes. Sci. Rep..

[26] Yin K, Paine ML (2017). Bicarbonate transport during enamel maturation. Calcif. Tissue Int..

[27] Zoccola D (2015). Bicarbonate transporters in corals point towards a key step in the evolution of cnidarian calcification. Sci. Rep..

[28] Romero MF, Fulton CM, Boron WF (2004). The SLC4 family of HCO 3—Transporters. Pflueg. Arch. Eur. J. Physiol..

[29] Lux SE, John KM, Kopito RR, Lodish HF (1989). Cloning and characterization of band 3, the human erythrocyte anion-exchange protein (AE1). Proc. Natl. Acad. Sci. U.S.A..

[30] Steck TL (1978). The band 3 protein of the human red cell membrane: A review. J. Supramol. Struct..

[31] Boedtkjer E, Praetorius J, Aalkjaer C (2006). NBCn1 (slc4a7) mediates the Na+-dependent bicarbonate transport important for regulation of intracellular pH in mouse vascular smooth muscle cells. Circ. Res..

[32] Abehsera S (2015). Binary gene expression patterning of the molt cycle: The case of chitin metabolism. PLoS ONE.

[33] Akiva-Tal A (2011). In situ molecular NMR picture of bioavailable calcium stabilized as amorphous CaCO3 biomineral in crayfish gastroliths. Proc. Natl. Acad. Sci. U.S.A..

[34] de Nooijer LJ, Spero H, Erez J, Bijma J, Reichart G-J (2014). Biomineralization in perforate foraminifera. Earth Sci. Rev..

[35] Duan X (2014). Ion channels, channelopathies, and tooth formation. J. Dent. Res..

[36] Calhoun S, Zou E (2016). Epidermal carbonic anhydrase activity and exoskeletal metal content during the molting cycle of the blue crab, *Callinectes sapidus*. J. Exp. Zool. A Ecol. Genet. Physiol..

[37] Gutknecht J, Bisson MA, Tosteson FC (1977). Diffusion of carbon dioxide through lipid bilayer membranes: Effects of carbonic anhydrase, bicarbonate, and unstirred layers. J. Gen. Physiol..

[38] Horst MN, Freeman JA (1993). The Crustacean Integument—Morphology and Biochemistry.

[39] Hampshire F, Horn DHS (1966). Structure of crustecdysone a Crustacean moulting hormone. Chem. Commun..

[40] Becker A, Ziegler A, Epple M (2005). The mineral phase in the cuticles of two species of Crustacea consists of magnesium calcite, amorphous calcium carbonate, and amorphous calcium phosphate. Dalton Trans..

[41] Bentov S (2012). Enamel-like apatite crown covering amorphous mineral in a crayfish mandible. Nat. Commun..

[42] Luquet G (2016). Calcium deposits in the crayfish, *Cherax quadricarinatus*: Microstructure versus elemental distribution. Microsc. Microanal..

[43] Abehsera S, Weil S, Manor R, Sagi A (2018). The search for proteins involved in the formation of crustacean cuticular structures. Hydrobiologia.

[44] Alper SL (2006). Molecular physiology of SLC4 anion exchangers. Exp. Physiol..

[45] Abehsera S (2017). MARS: A protein family involved in the formation of vertical skeletal elements. J. Struct. Biol..

[46] Gasteiger E (2005). Protein Identification and Analysis Tools on the ExPASy Server.

[47] Schultz J, Milpetz F, Bork P, Ponting CP (1998). SMART, a simple modular architecture research tool: Identification of signaling domains. Proc. Natl. Acad. Sci. U.S.A..

[48] Abehsera S (2018). CPAP3 proteins in the mineralized cuticle of a decapod crustacean. Sci. Rep..

[49] Gao Y (2015). Whole transcriptome analysis provides insights into molecular mechanisms for molting in *litopenaeus vannamei*. PLoS ONE.

[50] Ventura T (2009). Temporal silencing of an androgenic gland-specific insulin-like gene affecting phenotypical gender differences and spermatogenesis. Endocrinology.

[51] Sharabi O, Ventura T, Manor R, Aflalo ED, Sagi A (2013). Epidermal growth factor receptor in the prawn *Macrobrachium rosenbergii*: Function and putative signaling cascade. Endocrinology.

[52] Shpak N (2017). Short versus long double-stranded RNA activation of a post-transcriptional gene knockdown pathway. RNA Biol..

[53] Miculescu F (2020). Considerations and influencing parameters in EDS microanalysis of biogenic hydroxyapatite. J. Funct. Biomater..

[54] Ul-Hamid A (2018). A Beginners' Guide to Scanning Electron Microscopy.

